# Understanding racial/ethnic differences in e-cigarette outcome expectancies among early adolescents: findings from the Adolescent Brain Cognitive Development Study

**DOI:** 10.3389/fradm.2025.1556505

**Published:** 2025-05-11

**Authors:** John Tarantino, Tammy Chung, Nicole Kennelly, Shawn J. Latendresse, Margret Z. Powell, Carolyn E. Sartor

**Affiliations:** 1 Department of Psychiatry, Rutgers Robert Wood Johnson Medical School, Piscataway, NJ, United States,; 2 Institute for Health, Heath Care Policy and Aging Research, New Brunswick, NJ, United States,; 3 Department of Psychology and Neuroscience, Baylor University, Waco, TX, United States

**Keywords:** e-cigarette, adolescent, positive expectancies, negative expectancies, race, ethnicity

## Abstract

**Introduction:**

E-cigarette expectancies, which may differ by race/ethnicity, play a crucial role in shaping youth e-cigarette use. Observed differences by race/ethnicity, however, may reflect racial/ethnic variations in social determinants of health, such as socioeconomic status (SES). This study examined the extent to which race/ethnicity was uniquely associated with youths’ positive and negative e-cigarette expectancies, after adjusting for SES and neighborhood disadvantage, and individual, family, and peer risk factors.

**Methods:**

Analyses included 8,814 Black (15.0%), Latinx (22.8%), and White (62.2%) 12 to 14-year-old participants in the Adolescent Brain Cognitive Development Study. Applying a three-stage analytic approach, hierarchical regression analyses examined associations of positive and negative e-cigarette expectancies with race/ethnicity in three blocks, with age and gender in block 1, adding SES and neighborhood disadvantage in block 2, and individual, family, and peer risk factors in block 3.

**Results:**

Black and Latinx (relative to White) race/ethnicity and Latinx (relative to Black) race/ethnicity were associated with positive expectancies (*p* < 0.001) in blocks 1 and 2 but were non-significant in block 3. Black and Latinx (relative to White) race/ethnicity and Latinx (relative to Black) race/ethnicity were associated with lower negative expectancies (*p* < 0.001) in block 1, but were no longer significant after adding SES and neighborhood indicators in block 2. Perceived risk, perceived peer disapproval, and curiosity about e-cigarettes were associated with positive and negative expectancies.

**Discussion:**

The results highlight the importance of considering associations of race/ethnicity with e-cigarette expectancies in the context of social determinants and individual and interpersonal factors in e-cigarette prevention.

## 1 Introduction

The use of electronic cigarettes (e-cigarettes) among youth poses significant health risks, including possible progression to nicotine dependence and combustible cigarette use (1, 2), delays in brain development (3), and respiratory injury (4). According to the 2023 national Monitoring the Future survey, approximately one in seven 8th graders has tried e-cigarettes, with some modest variations in prevalence across racial/ethnic groups (5). By 12th grade, however, trends in prevalence diverge, with Black and Latinx youth reporting a higher lifetime prevalence of e-cigarette use relative to White youth (45% and 31% vs. 25%, respectively) (5). These findings underscore the importance of examining precursors to e-cigarette use, particularly among Black and Latinx youth at younger ages, to inform tailored prevention efforts.

Substance use outcome expectancies—beliefs about the anticipated effects (positive and negative) of substance use—consistently predict youth initiation of alcohol, cannabis, combustible cigarettes, and e-cigarette use (6–10). Expectancies exist even before an individual has direct experience with a substance (11) and can change after use has started (6, 12), which makes expectancies a key target for prevention. Positive e-cigarette expectancies include enjoyment or pleasure, reduced stress, appearing older, and improved social status, while negative e-cigarette expectancies involve, for example, concerns about potential addiction and adverse health effects (6, 13). Notably, a national survey of adolescents and young adults found that high positive e-cigarette expectancies were associated with an increased risk of e-cigarette initiation, while high negative e-cigarette expectancies protected against initiation of e-cigarette use (6).

A socio-ecological model of substance use initiation (14) suggests that multiple interconnected factors operating at individual, family, peer, and neighborhood levels can influence the development of e-cigarette expectancies and use among adolescents. Demographic characteristics such as gender and race/ethnicity may be associated with e-cigarette expectancies and use patterns. One study found that among youth with no vaping experience, girls had higher negative and positive e-cigarette expectancies relative to boys (15). Regarding race/ethnicity, research on expectancies is scarce. For example, higher positive e-cigarette expectancies have been observed among Black, relative to White and Latinx, high school students (15). In line with this finding, some studies have reported a higher lifetime prevalence of e-cigarette use among Black youth relative to their White peers (16, 17). One recent study found a higher prevalence of e-cigarette use among Latinx and White youth relative to Black youth, but found increasing rates of e-cigarette use among Black youth (18). In considering the implications for prevention, it is important to keep in mind that racial/ethnic differences in e-cigarette expectancies may be confounded by socioeconomic status (SES) and neighborhood factors, given the overrepresentation of Black and Latinx families in low-resource neighborhoods (19, 20).

We are unaware of prior research examining the association of SES with e-cigarette expectancies. However, some research suggests that, unlike combustible cigarettes, which are more prevalent in individuals from lower SES backgrounds (21), youth from higher SES households may be at greater risk of e-cigarette initiation regardless of race/ethnicity (22). It is important to note that the study by Hitchman et al. (21) focused on an older age group (18–24 years), which may limit its applicability to adolescent populations. Neighborhood conditions, including high exposure to e-cigarette products as a function of a high density of retailers in disadvantaged neighborhoods (23), may increase the risk of e-cigarette initiation (24) and potentially also e-cigarette expectancies. The current analyses examine unique associations of SES and neighborhood conditions with e-cigarette expectancies.

Beyond demographic and neighborhood factors, psychosocial factors at the individual, family, and peer levels of the socio-ecological model could contribute to e-cigarette expectancies and use in youth. Key predictors of e-cigarette use include personal attitudes toward use, peer influence, and parental use of e-cigarettes (25). For example, youth who perceive e-cigarettes as less harmful than combustible cigarettes are more likely to initiate use, underscoring the role of harm perceptions in shaping behaviors (25). Furthermore, exposure to family members and peers who use e-cigarettes significantly increases the likelihood of use (25). In addition, given that e-cigarette use itself can shape expectancies, leading to more favorable anticipated effects (6, 12) and the previously noted variation by race/ethnicity in the prevalence of e-cigarette use, it is critical to consider prior e-cigarette use when examining expectancies.

This secondary analysis of data from the Adolescent Brain Cognitive Development (ABCD) Study (26) explored demographic characteristics (race/ethnicity, gender identity, and age); SES and neighborhood conditions; and individual, family, and peer factors as predictors of e-cigarette positive and negative expectancies in youth. We addressed three research questions using a hierarchical regression modeling approach to evaluate the contribution of these constructs, entered in blocks, to predict e-cigarette expectancies above and beyond previously entered blocks of predictors. First, analyses examined how race/ethnicity, age, and gender related to expectancies. We tested the hypothesis, consistent with the relative prevalence of youth e-cigarette use by race/ethnicity (5), that the highest positive and lowest negative expectancies (indicative of greatest liability to e-cigarette use) would be reported by Black youth, followed by Latinx youth, with the lowest positive and highest negative expectancies observed in White youth. Second, indicators of SES and neighborhood disadvantage were added to the analysis to evaluate whether race/ethnicity continued to uniquely predict e-cigarette expectancies after adjusting for SES and neighborhood conditions (that is, to examine their potential confounding effects with race/ethnicity), given the disproportionate representation of Black and Latinx families in lower SES and disadvantaged neighborhoods (19, 20). Third, analyses examined whether race/ethnicity remained a unique predictor of e-cigarette expectancies after additionally adjusting for individual, family, and peer risk factors.

This three-step hierarchical regression analysis approach permitted evaluation of how structural factors (e.g., SES and neighborhood conditions) and individual and interpersonal (i.e., family and peer) risk factors contribute to observed race/ethnicity differences in e-cigarette expectancies. We hypothesized that associations of race/ethnicity with e-cigarette expectancies would be reduced after adjusting for SES and neighborhood conditions—social determinants of health that disproportionately impact people of color (27, 28). By examining these factors sequentially, in a hierarchical regression modeling approach, the current study aimed to provide a more nuanced understanding of the complex interplay between social determinants of health and youth perceptions of e-cigarette effects, offering novel insights into the correlates of e-cigarette health disparities.

## 2 Materials and methods

### 2.1 Data source

The ABCD Study (abcd.org) is an ongoing multi-site longitudinal study of adolescent health and cognitive development in the US (26). Details of the study design have been previously reported (29, 30).

Briefly, from 2016 to 2018, the study recruited youths (*N* = 11,875) aged 9–10 years, primarily through schools. Enrollment targets were derived using the National Center for Education Statistics and US Census data.

At annual assessments, youths and their primary caregivers (parents) completed a comprehensive assessment of mental and physical health, including a wide range of substance use-related factors, as well as cultural and environmental factors (31–33). Parents also reported demographic information (e.g., parental education level, youth race/ethnicity) and health information. Data for the current study were drawn mainly from Follow-up Year 3, release 5, the most recent data available at the time of analysis. In addition, data from baseline and prior follow-ups were used for specific variables, as detailed in the Measures section.

### 2.2 Participants

Our analysis included Black, Latinx, and White youth, the three largest racial/ethnic groups represented in the ABCD Study. Other racial/ethnic groups were excluded due to limited numbers, which would have resulted in insufficient statistical power. We used ABCD Study-defined race/ethnicity categories based on parent-reported youth ethnicity (Latinx/Hispanic or Non-Hispanic/Latinx) and youth race (in separate questions). Under this definition, all individuals endorsing Latinx/Hispanic were categorized as Latinx. Thus, “White” refers to Non-Latinx ethnicity and White race and “Black” as Non-Latinx Black/African American. Race/ethnicity was represented in the model using contrast coding, with one variable representing Black and Latinx race/ethnicity relative to White race/ethnicity and a second representing Latinx relative to Black race/ethnicity, thus allowing us to directly compare Black and Latinx youth. Youths’ self-reported gender identity, which was assessed with the question, “What is your current gender identity?” Response options were “boy,” “girl,” and “another gender (e.g., nonbinary).”

The analysis sample included 8,814 Black, Latinx, and White youths who completed all items in the ENDS Expectancies questionnaire at Follow-up 3 (*M*_age_ = 12.94, *SD* < 0.01; 53.07% self-identified as “boy,” 45.01% as “girl,” and 1.92% as “other gender;” 62.19% White, 15.03% Black, and 22.78% Latinx). The majority of parents reported an educational level of bachelor’s degree or higher (71.18%), and nearly half (48.88%) reported a household income of $100,000 or above. Detailed demographic characteristics of the sample are reported in Table 1.

### 2.3 Measures

#### 2.3.1 SES indicators

*The primary caregivers reported on their education level* with the question, “What is the highest grade or level of school you have completed or the highest degree you have received?” Consistent with prior ABCD publications (34), education level was collapsed into five categories: less than high school, high school, some college, bachelor’s degree, and post-bachelor’s degree. Education was represented in models with four dichotomous variables, using “some college” as the reference group.

*Household income* was categorized, consistent with prior ABCD publications (35), as low (less than $50,000), medium ($50,000–$99,999), and high ($100,000 or more), represented in the models with two dichotomous variables, using medium income as the reference group.

*The Area Deprivation Index (ADI)* was calculated by the ABCD Study by linking geocoded data from the parent-reported youth residence at baseline to census-tract data. The ADI is a widely used measure of neighborhood disadvantage, incorporating a variety of factors such as employment, household utilities, and housing values (36). ADI values are expressed as population-level (national) percentiles (ranging from 1 to 100), with higher values indicating greater disadvantage. For this study, the common approach of analyzing ADI quartiles was employed: 1 = ≤25th, 2 = 26th–50th, 3 = 51st–75th, and 4 = ≥76th percentiles. ADI was represented in models using three dichotomous variables, with the most advantaged (first ADI quartile) serving as the reference group.

#### 2.3.2 Individual, family, and peer risk factors

All risk factors other than e-cigarette use in the household were assessed via the youths’ report.

*Lifetime e-cigarette use* was assessed at baseline by asking, “Have you ever tried electronic cigarettes, vape pens, or e-hookah at any time in your life?” At subsequent follow-ups, youths were asked, “Have you had a puff of an e-cigarette, vape pen, or hookah since the last time we saw you?” These items were coded dichotomously (yes = 1, no = 0) and combined into a lifetime e-cigarette use variable (yes = 1, no = 0).

*E-cigarette users in the household* were assessed by asking the primary caregiver, “Did anyone use electronic nicotine or vaping products such as e-cigarettes, vape pens or Juuls inside the house when your child was home or in a vehicle your child was in?” (1 = “yes”, 0 = “no”).

*Perceived risk of regular e-cigarette use* was assessed by asking, “How much do you think people risk harming themselves (physically or in other ways) if they use e-cigarettes regularly?” The response options included “no risk,” “slight risk,” “moderate risk,” “great risk,” and “don’t know.” Due to the low percentage (as expected at this age) selecting “no risk” (2.62%), it was combined with “slight risk.” Three binary variables were included in the model: “no/low risk” (1 = “no/slight risk,” 0 = all other responses), “moderate risk,” and “don’t know.” “Great risk” served as the reference. This recoding addresses sparsely populated response categories and increases interpretability and statistical power by focusing on key distinctions in response options.

*Perceived peer disapproval of e-cigarette use* was assessed by the question, “How do you think your close friends feel (or would feel) about you using e-cigarettes regularly?” Possible responses included “not disapprove,” “disapprove,” “strongly disapprove,” and “don’t know.” For ease of interpretation, “disapprove” and “strongly disapprove” were combined and used as the comparison group. Two binary variables were included in the model: “not disapprove” (1 = “yes,” 0 = all other responses) and another indicating “don’t know” (1 = “yes,” 0 = all other responses). Combining these categories reduces sparse data for some response categories by focusing on meaningful distinctions and improving statistical power, reflecting the overall high degree of disapproval in this developmental period.

*Curiosity about e-cigarettes* was assessed with the question, “Have you ever been curious about using an electronic nicotine or vaping product?” Possible responses included “not at all curious,” “a little curious,” “somewhat curious,” “very curious,” and “don’t know.” Due to the small number of participants indicating any curiosity, responses were collapsed into a dichotomous variable: 1 = “a little curious,” “somewhat curious,” “very curious,” and “don’t know” vs. 0 = “not at all curious” (the reference group), to enhance interpretability, highlight meaningful distinctions, and improve statistical power.

#### 2.3.3 Outcomes

*E-cigarette outcome expectancies:* Positive and negative outcome expectancies regarding e-cigarette use were assessed at follow-up 3 using the eight-item revised Youth E-cigarette Outcome Expectancies measure (37). The measure queries four positive (e.g., feeling relaxed) and four negative (e.g., looking awkward) e-cigarette expectancies (rated 0 = unlikely to experience the effect and 9 = likely to experience the effect). After adjustment for measurement equivalence with respect to race/ethnicity, sex assigned at birth, and the intersection of race/ethnicity and sex [see Chung et al. (38) for details on measurement equivalence methods], the positive and negative expectancy scales yielded continuous scores for each subscale. These scores were used as the outcome measures in the analysis.

### 2.4 Analysis plan

Analyses were conducted in SAS version 8.3 using SAS SURVEY procedures to accommodate the ABCD Study’s complex sample ascertainment design. Analyses accounted for clustering within the family and the study site. For the hierarchical regression analyses, standard ABCD Study sample weights were applied per ABCD Study analysis guidelines (39). Prior to finalizing regression analyses, collinearity diagnostics were conducted; they did not support the exclusion of any explanatory variables. Regression analyses (SURVEYREG) fitted a general linear model to the data in three steps (or three blocks), separately for positive (Table 2) and negative (Table 3) e-cigarette expectancies. The SURVEYREG procedure calculates coefficient estimators (standardized coefficients are reported) using generalized least squares estimation via elementwise regression (40) and uses Taylor series to estimate the sampling errors of estimators (40). The first block included only race/ethnicity, gender, and age. Race/ethnicity was represented in the model (and subsequent models) using contrast coding, with two variables, one representing the contrast between Black and Latinx relative to White youth and one representing the contrast between Latinx and Black youth. The second block added SES and neighborhood indicators: parental education, household income, and ADI. The third block added the individual, family, and peer risk factors (e.g., lifetime e-cigarette use, perceived risk of regular use of e-cigarettes). Note that, as hierarchical regression analysis was used to disaggregate unique explanatory contributions of elements within a model testing a single, albeit complex, hypothesis, there was no need to account for possible Type 1 error inflation, as would be the case with multiple testing.

## 3 Results

Note that adjustment for measurement equivalence in the expectancies subscales resulted in continuous factor scores with a range that included negative values. The mean adjusted positive expectancies score for the full sample was 0.04 (*SE* = 0.01, range: −1.06 to 3.04), while the mean adjusted negative e-cigarette expectancy score for the full sample was 0.02 (*SE* = 0.01, range: −2.58 to 1.64). Mean positive and negative expectancy scores are reported by race/ethnicity in Table 4.

### 3.1 Block 1: race/ethnicity, gender, and age

#### 3.1.1 Positive e-cigarette expectancies

In block 1 (Table 2), Black/Latinx race/ethnicity (relative to White race/ethnicity) was associated with significantly lower positive expectancies [coefficient = −0.073 (standardized value), *p* = 0.001]. Latinx race/ethnicity, when compared to Black race/ethnicity, was associated with significantly higher positive expectancies (coefficient = 0.129, *p* < 0.001). There was no significant difference in positive expectancies between youths identifying as girls vs. those identifying as boys. However, individuals identifying as another gender reported significantly higher positive expectancies than those who identified as boys (coefficient = 0.436, *p* < 0.001). The R^2^ value for block 1 was 0.019, indicating that the model explained approximately 1.9% of the variance in positive expectancies.

#### 3.1.2 Negative e-cigarette expectancies

In block 1 (Table 3), identifying as Black or Latinx (relative to White) was associated with lower negative e-cigarette expectancies (coefficient = −0.102, *p* < 0.001), while identifying as Latinx (relative to Black) was associated with higher negative expectancies (coefficient = 0.099, *p* = 0.005). There was no significant difference in negative e-cigarette outcome expectancies between youths identifying as girls and boys. However, individuals identifying as another gender reported significantly lower negative expectancies compared to boys (coefficient = −0.278, *p* < 0.001). The R^2^ value for block 1 was 0.006, reflecting 0.6% of the variance in negative expectancies.

### 3.2 Block 2: addition of SES and neighborhood factors

#### 3.2.1 Positive e-cigarette expectancies

In block 2 (Table 2), identifying as Black or Latinx (relative to White race/ethnicity) was associated with lower positive e-cigarette expectancies (coefficient = −0.058, *p* = 0.033). Identifying as Latinx (relative to Black) was associated with higher positive expectancies (coefficient = 0.122, *p* = 0.001). In addition, the second ADI quartile was associated with lower positive e-cigarette expectancies relative to the lowest ADI quartile (i.e., most advantaged; coefficient = −0.056, *p* = 0.021). SES indicators were not significant. The addition of SES and neighborhood variables in block 2 accounted for an additional 0.20% of the explained variance in positive expectancies.

#### 3.2.2 Negative e-cigarette expectancies

In block 2 (Table 3), neither identifying as Black or Latinx (relative to White) nor identifying as Latinx (relative to Black) was significantly associated with negative e-cigarette expectancies. Relative to the lowest ADI quartile, the highest ADI quartile (most disadvantaged) was associated with lower negative e-cigarette expectancies (coefficient = −0.201, *p* < 0.001). Further, primary caregiver education level of high school or lower (relative to some college) was associated with lower negative e-cigarette expectancies (*p*s < 0.01). In addition, household income >$100,000 (vs. medium household income) was associated with high negative e-cigarette expectancies (*p* = 0.012). The addition of the SES and neighborhood variables in block 2 accounted for an additional 1.11% of the explained variance in negative outcome expectancies.

### 3.3 Block 3: addition of individual, family, and peer risk factors

#### 3.3.1 Positive e-cigarette expectancies

In block 3 (Table 2), which accounted for individual, family, and peer risk factors related to e-cigarette use, neither of the race/ethnicity variables—Black/Latinx (compared to White) and Latinx (compared to Black)—was significantly associated with positive expectancies (coefficient = −0.025, *p* = 0.338; coefficient = 0.069, *p* = 0.067, respectively). SES indicators were non-significant, but ADI was significant; relative to quartile 1 (most advantaged), all three quartiles were associated with lower positive expectancies (second quartile: coefficient = −0.065, *p* = 0.005; third quartile: coefficient = −0.067, *p* = 0.032; fourth quartile: coefficient = −0.081, *p* = 0.028). Among the predictors added in block 3, e-cigarette use in the household, perceived risk of regular e-cigarette use (no/slight to moderate risk), perception that peers do not disapprove of e-cigarette use, and report of being at least a little curious about e-cigarettes were each positively associated with positive e-cigarette expectancies (*p*s < 0.001). The addition of variables in block 3 accounted for an additional 11.4% of the explained variance in positive expectancies.

#### 3.3.2 Negative e-cigarette expectancies

In block 3 (Table 3), after including possible individual, family, and peer factors related to e-cigarette use, neither of the race/ethnicity variables was significantly related to negative expectancies. All SES indicators were non-significant except for the following: having less than a high school education (compared to some college) was associated with lower negative expectancies (coefficient = −0.213, *p* < 0.001), as was completing high school (coefficient = −0.108, *p* = 0.022). In addition, being in the fourth ADI quartile (i.e., most disadvantaged) compared to the first quartile (most advantaged) was associated with lower negative expectancies (coefficient = −0.135, *p* = 0.001). Among the predictors added in block 3, perceived risk of regular e-cigarette use (no/slight to moderate risk, don’t know), perception that peers do not disapprove of e-cigarette use (and don’t know), and report of being at least a little curious were each uniquely negatively associated with negative e-cigarette expectancies (*p*s < 0.001). The added variables in block 3 accounted for an additional 10.1% of the explained variance in negative e-cigarette expectancies.

## 4 Discussion

### 4.1 Race/ethnicity and e-cigarette expectancies: results of hierarchical regression analyses

The present study examined associations of race/ethnicity with positive and negative e-cigarette outcome expectancies in adolescents in the context of SES, neighborhood conditions, and individual, family, and peer risk factors. We found partial support for each of our three hypotheses regarding race/ethnicity differences and the extent to which socioeconomic disadvantage indicators may confound associations and the unique contributions of individual, family, and peer risk factors.

Our hypothesis—that Black and Latinx youth would have higher positive expectancies than White youth before adjusting for socioeconomic and neighborhood factors—was based on racial/ethnic differences in e-cigarette use prevalence from the Monitoring the Future study (5) and prior research showing higher positive expectancies among racial/ethnic minority youth than White youth (15). We expected positive expectancies to follow the same pattern as prior research. However, we found the opposite: Black and Latinx youths reported lower positive expectancies than White youths, with Latinx youths showing higher expectancies than Black youths.

Notably, a key methodological distinction that could help explain the difference in results across studies is that Morean et al. (15) categorized participants into broad racial/ethnic groups, without directly comparing Black and Latinx youth. In contrast, our study separately examined non-Latinx White, non-Latinx Black, and Latinx youth, making it one of the first studies to directly compare e-cigarette expectancies between Black and Latinx adolescents. This distinction is critical, as it allows for a more nuanced understanding of how expectancies differ within minoritized groups rather than solely in contrast to White youth.

In addition, differences in results across this study and that by Morean et al. (15) might also reflect developmental differences in exposure to and experience with e-cigarettes between high school-aged youth (15) and the younger sample (ages 12–14) studied here. The types of e-cigarette expectancies examined by Morean et al. (15), and in this study also differed, although measurement equivalence by race/ethnicity was used in both studies.

Our hypothesis that distinctions by race/ethnicity in positive expectancies would be reduced following the addition of SES and neighborhood factors was not supported. The retention of significant associations between race/ethnicity and positive expectancies suggests that race/ethnicity differences were not simply a marker for the effects of socioeconomic disadvantage on positive expectancies. The reduction of race/ethnicity differences to non-significance after family, peer, and individual risk factors were included suggested that variation by race/ethnicity was attributable, at least in part, to these risk factors.

In contrast to the results for positive expectancies, we found support for our hypothesis that prior to considering socioeconomic and neighborhood factors, negative expectancies would be lower among Black and Latinx youth relative to White youth, and Latinx youth relative to Black youth. In further support of our hypotheses, negative e-cigarette expectancies among Black and Latinx youths were attributable at least in part to social determinants of health (i.e., SES, neighborhood factors). As to why this result was found for negative, but not positive expectancies, we can only speculate. It is possible that negative expectancies are more strongly shaped by structural factors, such as exposure to anti-tobacco messaging, school policies, and community norms that discourage substance use. In contrast, positive expectancies may be more influenced by direct individual and peer experiences with e-cigarettes. Taken together, these findings suggest that observed racial/ethnic differences in e-cigarette expectancies are attributable in part to SES and neighborhood conditions, in the case of negative expectancies, and to individual, family, and peer risk factors in the case of positive expectancies.

### 4.2 Co-occurrence of positive and negative e-cigarette expectancies

Our findings indicated that racial/ethnic groups with high positive expectancies also tended to report high negative expectancies, a pattern that may seem counterintuitive. This pattern of endorsement suggests that youth who recognize the potential benefits of e-cigarette use, such as stress relief or social acceptance, are also aware of its potential harms, including addiction or respiratory issues. This phenomenon is consistent with prior research showing that adolescents can simultaneously hold both risk-promoting and risk-deterring beliefs about substance use (41). This finding also raises the possibility that the distinction between anticipated effects characterized as “positive” or “negative” may not be strong among youth at this age, and thus, the measure may be capturing the degree to which effects of e-cigarettes more generally are expected.

### 4.3 Associations of socioeconomic status and neighborhood conditions with expectancies

The results from our hierarchical regression models reveal complex relationships of SES and neighborhood disadvantage with expectancies, including distinctions between positive and negative expectancies in these relationships. For instance, whereas parental education was unrelated to positive expectancies—either in the presence or absence of individual, family, and peer risk factors—parental education at the high school level or below was associated with lower negative expectancies, even after accounting for individual, family, and peer risk factors. One possibility for the specificity of this association to negative expectancies is that education regarding the harms of e-cigarette use, which may be more accessible to highly educated populations, would impact negative expectancies to a greater degree than positive expectancies. With respect to neighborhood disadvantage, living in any neighborhood conditions below the most advantaged was associated with lower positive expectancies after accounting for individual, family, and peer risk factors, whereas only living in the most disadvantaged neighborhood conditions was associated with lower negative expectancies. The association of neighborhood disadvantage with lower e-cigarette expectancies, in general, might reflect more limited exposure and access to e-cigarettes for youth who reside in more disadvantaged neighborhoods (42). Notably, the relation of neighborhood conditions (e.g., access and availability) and SES with e-cigarette expectancies and e-cigarette use will likely change with age, a dynamic that can be captured in future ABCD data collections (24).

### 4.4 Individual, family, and peer risk factors in relation to expectancies

At the individual, family, and peer levels, key predictors of both positive and negative e-cigarette expectancies included e-cigarette use in the household, curiosity about e-cigarettes, perceived risk, and perception of peer disapproval of e-cigarette use. These findings are consistent with previous studies that highlight the role of peer influence and parental use as contributors to adolescent attitudes toward e-cigarettes (25, 43, 44). The results underscore the importance of the social environment in shaping e-cigarette expectancies, for example, beliefs that their peers do not disapprove of e-cigarette use leading to higher positive expectancies, and the need to target these social factors in prevention efforts.

### 4.5 Limitations

Several limitations need to be considered when interpreting study findings. First, there was heterogeneity within racial/ethnic categories of “Black,” “Latinx”, and “White” that are not addressed here. Second, the analyses were cross-sectional and, therefore, only capture concurrent associations. Longitudinal data would be necessary to examine how e-cigarette expectancies change over time. Third, while these analyses included a wide range of demographic and psychosocial factors, there are other unmeasured risk and protective factors that contribute to e-cigarette expectancies.

### 4.6 Conclusions

Findings from the present study highlight the importance of considering differences by race/ethnicity in e-cigarette expectancies in the context of social determinants of health from a developmental perspective, and suggest intervention targets at multiple levels of influence. The endorsement of positive expectancies by Black, Latinx, and White middle-school-aged youths in this sample did not track with the patterns by race/ethnicity in the prevalence of e-cigarette use or expectancies in the high school years (5, 15). Together, the results suggest that as expectancies evolve across developmental periods, distinctions across racial/ethnic groups also shift. Critically, our hierarchical regression analysis approach revealed that most associations of race/ethnicity with e-cigarette expectancies were reduced after adjusting for social determinants of health, specifically SES and neighborhood disadvantage. Finally, the consistency across positive and negative expectancies in the relevance of perceived risk, perceived peer disapproval, and curiosity about e-cigarettes suggest multiple potential targets for early prevention efforts.

## Figures and Tables

**TABLE 1:** Sample demographic characteristics by race/ethnicity.

Variable	Total		White		Black		Latinx		% missing
*N* (%)	8,814 (100.00)		5,481 (62.19)		1,325 (15.03)		2,008 (22.78)		0.00
	*N* or *M*	% or *SE*	*N* or *M*	% or *SE*	*N* or *M*	% or *SE*	*N* or *M*	% or *SE*	%
Age	12.94	>0.01	12.96	0.01	12.93	0.02	12.90	0.02	0.00
**Gender identity**									**1.41**
Girl	3,911	45.01	2,393	44.18	640	49.12	878	44.57	
Boy	4,612	53.07	2,907	53.66	649	49.81	1,056	53.60	
Other	167	1.92	117	2.16	14	1.07	36	1.83	
**Caregiver education level**									**0.01**
Less than high school	381	4.32	36	0.66	80	6.04	265	13.02	
High school	1,001	11.36	253	4.62	377	27.72	381	18.97	
Some college	1,158	13.14	645	11.77	218	16.47	295	14.69	
Bachelor’s degree	2,545	28.88	1,970	33.94	189	14.27	386	19.22	
Post-bachelor’s degree	3,728	42.30	2,577	47.02	470	35.50	681	33.50	
**Yearly household income**									**1.65**
<$50,000	2,169	25.02	522	9.63	775	60.50	872	44.40	
$50,000–99,999	2,263	26.10	1,360	25.07	308	24.04	595	30.30	
>$100,000	4,237	48.88	3,542	65.30	198	15.46	497	25.31	
**Area Deprivation Index**									**6.89**
First quartile	2,900	35.34	2,205	42.83	130	10.98	565	30.13	
Second quartile	2,951	35.96	1,979	38.44	218	18.41	754	40.21	
Third quartile	1,281	15.61	675	13.11	275	23.23	331	17.65	
Fourth quartile	1,075	13.10	289	5.61	561	47.38	225	12.00	

M, mean SE, standard error.

**TABLE 2:** Hierarchical regression model predicting adjusted positive e-cigarette expectancies.

Variable	Block 1		Block 2		Block 3	
	Coefficient	*p*	Coefficient	*p*	Coefficient	*p*
Age	0.011	<0.001	0.011	<0.001	0.007	<0.001
**Gender identity**						
Girl	0.033	0.114	0.033	0.105	0.035	0.080
Other gender identity	0.436	<0.001	0.439	<0.001	0.252	<0.001
**Race/ethnicity**						
Black/Latinx vs. White	−0.073	0.001	−0.058	0.033	−0.025	0.338
Latinx vs. Black	0.129	<0.001	0.122	0.001	0.069	0.067
**Caregiver education level**						
Less than high school			0.015	0.808	0.062	0.298
High school			−0.009	0.830	0.015	0.723
Bachelor’s degree			−0.027	0.465	0.024	0.487
Post-bachelor’s degree			0.012	0.739	0.051	0.122
**Yearly household income**						
<$50,000			−0.058	0.070	−0.059	0.055
>$100,000			−0.027	0.284	0.012	0.610
**Area Deprivation Index**						
Second quartile			−0.056	0.021	−0.065	0.005
Third quartile			−0.031	0.345	−0.067	0.032
Fourth quartile			−0.040	0.286	−0.081	0.028
**Lifetime e-cigarette use**						
Yes					0.137	0.103
**Use of e-cigarettes in home**						
Yes					0.136	0.001
**Perceived risk of regular e-cigarette Use**						
No risk/slight risk					0.313	<0.001
Moderate risk					0.333	<0.001
Don’t know					0.049	0.300
**Perceived peer disapproval**						
Not disapprove					0.315	<0.001
Don’t know					0.009	0.845
**Curiosity about e-cigarettes**						
At least a little curious					0.448	<0.001
Model R^2^	0.019		0.021		0.135	
ΔR^2^ (relative to prior model)			0.002		0.114	

Hierarchical regression analyses were conducted to quantify potential changes in the magnitude of associations as additional sets of variables were added to the model. Model coefficients are standardized. Block 1 included only demographic variables, such as race/ethnicity, gender identity, and age. Block 2 incorporated SES and neighborhood conditions and block 3 added potential individual, family, and peer factors related to e-cigarette use. The sample size for the analyses was 8,814.

**TABLE 3:** Hierarchical regression model predicting adjusted negative e-cigarette expectancies.

Variable	Block 1		Block 2		Block 3	
	Coefficient	*p*	Coefficient	*p*	Coefficient	*p*
Age	−0.003	0.024	−0.003	0.013	−0.001	0.685
**Gender identity**						
Girl	0.012	0.571	0.011	0.612	−0.010	0.642
Other gender identity	−0.278	<0.001	−0.268	<0.001	−0.133	0.047
**Race/ethnicity**						
Black/Latinx vs. White	−0.102	<0.001	0.002	0.933	0.011	0.673
Latinx vs. Black	0.099	0.005	0.039	0.298	0.038	0.326
**Caregiver education level**						
Less than high school			−0.197	<0.001	−0.213	<0.001
High school			−0.123	0.008	−0.108	0.022
Bachelor’s degree			−0.013	0.738	−0.054	0.145
Post-bachelor’s degree			−0.054	0.134	−0.072	0.040
**Yearly household income**						
<$50,000			0.006	0.845	0.018	0.583
>$100,000			0.068	0.012	0.032	0.220
**Area Deprivation Index**						
Second quartile			−0.012	0.630	0.003	0.892
Third quartile			−0.043	0.194	−0.013	0.704
Fourth quartile			−0.201	<0.001	−0.135	0.001
**Lifetime e-cigarette use**						
Yes					−0.131	0.063
**Use of e-cigarettes in home**						
Yes					−0.071	0.088
**Perceived risk of regular e-cigarette use**						
No risk/slight risk					−0.589	<0.001
Moderate risk					−0.383	<0.001
Don’t know					−0.580	<0.001
**Perceived peer disapproval**						
Not Disapprove					−0.283	<0.001
Don’t know					−0.220	<0.001
**Curiosity about e-cigarettes**						
At least a little curious					−0.121	<0.001
Model R^2^	0.006		0.017		0.118	
ΔR^2^ (relative to prior model)			0.011		0.101	

Hierarchical regression analyses were conducted to quantify potential changes in the magnitude of associations as additional sets of variables were added to the model. Model coefficients are standardized. Block 1 included only demographic variables, such as race/ethnicity, gender identity, and age. Block 2 incorporated SES and neighborhood conditions and block 3 added potential individual, family, and peer factors related to e-cigarette use. The sample size for the analyses was 8,814.

**TABLE 4:** Individual, family, and peer risk factors by race/ethnicity.

Variable	Total		White		Black		Latinx		% missing
*N* (%)	8,814 (100.00)		5,481 (62.19)		1,325 (15.03)		2,008 (22.78)		0.00
	*N* or *M*	% or *SE*	*N* or *M*	% or *SE*	*N* or *M*	% or *SE*	*N* or *M*	% or *SE*	%
**Lifetime e-cigarette use**									**0.00**
Yes	195	2.21	74	1.35	11	0.83	49	2.44	
No	8,619	97.79	5,407	98.66	1,314	99.17	1,959	97.56	
**Use of e-cigarettes in home**									**5.93**
Yes	655	7.43	429	8.08	83	7.23	143	7.79	
No	8,159	92.57	4,879	91.92	1,065	92.77	1,692	92.21	
**Perceived risk of regular e-cigarette use**									**0.05**
No risk	231	2.62	59	1.08	108	8.16	64	3.19	
Slight risk	751	8.52	411	7.50	141	10.66	199	9.91	
Moderate risk	2,344	26.61	1,524	27.82	281	21.24	539	26.84	
Great risk	5,004	56.80	3,263	59.55	667	13.33	1,074	53.49	
Don’t know	480	5.45	222	4.05	126	9.52	132	6.57	
**Perceived peer disapproval**									**0.05**
Not disapprove	297	3.37	158	2.88	58	4.38	81	4.03	
Disapprove	1,963	22.28	1,088	19.86	370	27.97	505	25.15	
Strongly disapprove	6,087	69.09	4,016	65.98	791	59.79	1,280	63.75	
Don’t know	463	5.26	217	3.96	104	7.86	142	7.07	
**Curiosity about e-cigarettes**									**5.89**
Not at all curious	7,256	87.47	4,566	88.59	1,100	87.09	1,590	84.66	
A little curious	698	8.41	420	8.15	90	7.13	188	10.01	
Somewhat curious	180	2.17	106	2.06	29	2.30	45	2.54	
Very curious	41	0.49	15	0.29	14	1.11	12	0.64	
Don’t know	97	1.17	38	0.74	24	1.90	35	1.86	
No response	23	0.28	9	0.17	6	0.48	8	0.43	
**Adjusted positive outcome expectancy score**									**0.00**
	0.04	0.01	0.06	0.01	−0.09	0.03	0.05	0.02	
**Adjusted negative outcome expectancy score**									**0.00**
	0.02	0.01	0.01	0.01	−0.01	0.03	−0.01	0.02	

*M*, mean; *SE*, standard error.

e-cigarette positive and negative expectancy scores were adjusted for possible measurement bias by race/ethnicity and sex.

## Data Availability

The data analyzed in this study is subject to the following licenses/restrictions: Data used in the preparation of this article were obtained from the Adolescent Brain Cognitive Development (ABCD) Study (https://abcdstudy.org), held in the NIMH Data Archive (NDA). A full list of supporters is available at https://abcdstudy.org/federal-partners.html. A listing of participating sites and a complete listing of the study investigators can be found at https://abcdstudy.org/consortium_members/. ABCD consortium investigators designed and implemented the study and/or provided data but did not necessarily participate in the analysis or writing of this report. Requests to access these datasets should be directed to https://abcdstudy.org.
